# Academical Proceedings, National and Foreign

**Published:** 1822-04

**Authors:** 


### ACADEMICAL PROCEEDINGS,

### NATIONAL AND FOREIGN.

### 1. ROYAL SOCIETY OF LONDON.

AT the meeting of the 7th of March, Sir THOMAS LAWRENCE, president of the Royal Academy, was admitted a fellow of the Society.

An interesting paper, by Mr. SCORESBY, was then read, on the Development of Magnetic Power by Percussion in thin Bars of Iron. When the percussion is made on a small iron bar, the magnetic effects are but slight; but, by following a similar process with a common poker, the development of magnetic power which ensues is very considerable. It is a curious fact, that, when the bar has been thus rendered magnetic, if a single stroke be given on one of its extremities, or on the opposite side to that which had been formerly subjected to percussion, the magnetic power disappears.

By means of percussion alone, Mr. Scoresby has succeeded in making magnets possess considerable lifting power. The memoir is replete with interesting and curious observations.

Mr. Richard Philips, the eminent chemist, and editor of the Annals of Philosophy, was elected a fellow of the Royal Society, at the meeting of the 14th.

Messrs. Stoddart and Farraday presented a memoir on the different Alloys of Steel with Silver, Platinum, Palladium, Osmium, Cromium, &c. The experiments made by these gentlemen, on a small scale, in the laboratory of the Royal Institution, on the same subject, have been laid before the public. The authors, being anxious to ascertain how far the same results might be obtained when the experiments were made on a sufficiently extensive scale to render them available for practical purposes, undertook those which they have detailed to the Society in the present memoir, and which seem to be most satisfactory.

This paper was continued at the meeting of the 21st instant.

### 2. GEOLOGICAL SOCIETY OF LONDON.

The anniversary general meeting of this flourishing and highly useful Society was held on the 1st day of February, when the following members were elected officers of the Society for the ensuing year:—

*President.*—William Babington, M.D. F.R.S.

*Vice-Presidents.*—Rev. William Buckland, F.R.S. Wm. Haseldine Pepys, Esq. F.R.S. Henry Warburton, Esq. F.R.S. and William Hyde Wollaston, M.D. F.R.S.

*Secretaries.*—William Henry Filton, M.D. F.R.S. and Mr. Thomas Webster.

*Foreign Secretary.*—Henry Heuland, Esq.

*Council.*—Hon. Henry Grey Bennet, M.P. F.R.S. Arthur Aikin, Esq. F.L.S. John Bostock, M.D. F.R.S. and F.L.S. Henry James Brooke, Esq. F.R.S. and F.L S. Daniel Moore, Esq. F.R.A.S. and F.L.S. George Belias Greenough, Esq. F.R.S. and F.L.S. Major Thomas Colby, LL.D. F.R.S. L. and E. Augustus Bozzi Granville, M.D. F.R.S. and F.L.S. Peter M. Roget, M.D. F.R.S. Thomas Smith, Esq. F.R.S. and F.L.S. Charles Stokes, Esq. F.R.A.S. and F.L.S. and Philip Barker Webb, Esq.

Mr. Thomas Webster, keeper of the museum, and draughtsman.

### 3. MEDICAL SOCIETY OF LONDON, BOLT-COURT.

On the 8th of March was held the anniversary of this Society, when the following officers, and the council, were chosen for the ensuing year:—

*President.*—Dr. Uwins.

*Vice-Presidents.*—Dr. John Mason Good, Dr. John Gordon Smith, Mr. Andree, and Mr. Pettigrew.

*Treasurer.*—Mr. Andree.

*Secretaries.*—Mr. Pettigrew and Mr. Callaway.

*Secretary for Foreign Correspondence.*—Dr. Henry Blegborough.

*Registrar.*—Mr. James Field.

*Council.*—Doctors Walshman, Clutterbuck, Blegborough, Copland, Blicke, Martin, Stewart, Ley, Armstrong, and Harrison; Messrs. Lowdell, Winder, Grainger, Sutleffe, Drysdale, Box, Johnston, Dunlap, Brien, Lake, Thrasycles Clarke, Brown, Burrows, Tarratt, Thomas Clarke, Rees, Kingdon, W. T. Ward, Austen, Huggett, Cartwright, Morgan, Sawrey, and Taylor.

To deliver the oration in March 1823,—Mr. Grainger.

The oration was by Dr. Copland, on the Laws of Electricity, as connected with inorganic and animate Existences. The room was exceedingly crowded; and the dinner, which took place at the Globe Tavern, was more numerously attended than has been the case for many years, the number of persons present being eighty-five. The president took occasion to congratulate the Society on its recently improved, and still rapidly improving, condition; and the evening was spent much to the gratification of both members and visitors.

4. THE ACADEMICAL SOCIETY OF THE LOWER LOIRE

Has proposed a prize, consisting of a gold medal, value 300 francs, for the best answer to questions respecting the yellow-fever. It is required to trace its origin; to specify its causes and nature; to describe the state of the atmosphere, and local circumstances, where it prevails; to notify its identity, or otherwise, with similar fevers in Europe, &c.; to distinguish whether it be complicated with any other malady. There is also a second subject, relating to the means for preventing its spreading, the proper modes of quarantine, &c.

The memoirs to be sent, post free, to the Secretary of the Society, before the 1st of May, 1822. Each to bear a motto, with a repetition in a sealed paper, containing, as usual, the author's name and address.

5. THE ROYAL SOCIETY OF MEDICINE AT MARSEILLES

Has proposed the following questions:—1. To [illegible]rmine the structure and functions of the spinal marrow. 2. To describe the nature, causes, symptoms, and treatment, of the diseases by which the spinal marrow is affected. It is desired that clinical observations and pathological anatomy should be made the principal objects of the memoirs. They may be written in Latin or French. The extent of time allowed is till July 1822, and the prize a gold medal.

☞ The Editor of the London Medical and Physical Journal has withdrawn his name from the list of members of the Medico-Chirurgical Society of London.

